# Factors influencing exclusive breastfeeding rates until 6 months postpartum: the Japan Environment and Children’s Study

**DOI:** 10.1038/s41598-021-85900-4

**Published:** 2021-03-25

**Authors:** Hitomi Inano, Mariko Kameya, Kyoko Sasano, Kenta Matsumura, Akiko Tsuchida, Kei Hamazaki, Hidekuni Inadera, Tomomi Hasegawa, The Japan Environment and Children’s Study (JECS) Group, Michihiro Kamijima, Shin Yamazaki, Yukihiro Ohya, Reiko Kishi, Nobuo Yaegashi, Koichi Hashimoto, Chisato Mori, Shuichi Ito, Zentaro Yamagata, Takeo Nakayama, Hiroyasu Iso, Masayuki Shima, Youichi Kurozawa, Narufumi Suganuma, Koichi Kusuhara, Takahiko Katoh

**Affiliations:** 1 Department of Maternal Nursing, Graduate School of Medicine and Pharmaceutical Science for Education, University of Toyama, Toyama City, 930-0194 Japan; 2 Division of Maternal Nursing, Graduate School of Medicine and Pharmaceutical Science for Research, University of Toyama, Toyama City, 930-0194 Japan; 3 Toyama Regional Center for JECS, University of Toyama, Toyama City, 930-0194 Japan; 4 Department of Public Health, Faculty of Medicine, University of Toyama, Toyama City, 930-0194 Japan; 5 Graduate School of Medical Sciences Department of Occupational and Environmental Health, Nagoya City University, 1 Kawasumi, Mizuho-cho, Mizuho-ku, Nagoya, Aichi 467-8601 Japan; 6 National Institute for Environmental Studies, Tsukuba, Japan; 7 National Center for Child Health and Development, Tokyo, Japan; 8 Hokkaido University, Sapporo, Japan; 9 Tohoku University, Sendai, Japan; 10 Fukushima Medical University, Fukushima, Japan; 11 Chiba University, Chiba, Japan; 12 Yokohama City University, Yokohama, Japan; 13 University of Yamanashi, Chuo, Japan; 14 Kyoto University, Kyoto, Japan; 15 Osaka University, Suita, Japan; 16 Hyogo College of Medicine, Nishinomiya, Japan; 17 Tottori University, Yonago, Japan; 18 Kochi University, Nankoku, Japan; 19 University of Occupational and Environmental Health, Kitakyushu, Japan; 20 Kumamoto University, Kumamoto, Japan

**Keywords:** Nutrition, Public health, Health care, Health policy

## Abstract

This research aimed to examine the efficacy of the early initiation of breastfeeding within 1 h of birth, early skin-to-skin contact, and rooming-in for the continuation of exclusive breastfeeding until 6 months postpartum. The research used data from the Japan Environment and Children’s Study (JECS), a nationwide government-funded birth cohort study. A total of 80,491 mothers in Japan between January 2011 and March 2014 who succeeded or failed to exclusively breastfeed to 6 months were surveyed in JECS. Multiple logistic regression model was used to analyse the data. The percentage of mothers who succeeded in exclusively breastfeeding to 6 months is 37.4%. Adjusted odds ratios were analysed for all 35 variables. Early initiation of breastfeeding (adjusted odds ratio [AOR]: 1.455 [1.401–1.512]), early skin-to-skin contact (AOR: 1.233 [1.165–1.304]), and rooming-in (AOR: 1.567 [1.454–1.690]) affected continuation of exclusive breastfeeding. Regional social capital (AOR: 1.133 [1.061–1.210]) was also discovered to support the continuation of breastfeeding. In contrast, the most influential inhibiting factors were starting childcare (AOR: 0.126 [0.113–0.141]), smoking during pregnancy (AOR: 0.557 [0.496–0.627]), and obese body type during early pregnancy (AOR: 0.667 [0.627–0.710]).

## Introduction

Breastfeeding has been reported to lower a child’s risk of infectious disease and allergic illness while also being beneficial to the health of the mother^1^. Given that it contributes to the lifelong health of both mother and child, breastfeeding is widely encouraged throughout the world. The United Nations Children’s Fund (UNICEF), the World Health Organization (WHO), and the American Academy of Pediatrics all recommend exclusive breastfeeding (EBF)^2,3^ for the first 6 months postpartum. During EBF, breast milk must be the infant’s only intake, without additional food or drink, including water.

According to surveys conducted by the Ministry of Health, Labour and Welfare in Japan, more than 90% of pregnant women hope to breastfeed their children. Nevertheless, only 50% of mothers are able to continue EBF with their children up to even 3 months postpartum^4^.

Factors influencing breastfeeding rates include social demographic factors such as the mother’s age^5^, race^6^, level of education^7^, smoking habits^8^ and presence or absence of obesity^9^. The list of “Ten Steps to Successful Breastfeeding” proposed by the WHO and UNICEF contains additional items such as early initiation of breastfeeding (within 30 min of birth), early skin-to-skin contact (Step 4) and 24-h rooming-in (Step 7). Rooming-in entails placing the infant in a stand-alone cot by the bedside of the mother or bed-sharing by attached side-car crib as opposed to keeping the infant in a hospital nursery or separate room (in case of home-deliveries). Large-scale surveys investigating whether these kinds of early postpartum mother–child care practices affect EBF up to 6 months postpartum are rare worldwide and do not exist within Japan.

Using data from the Japan Environment and Children’s Study (JECS), the present study aims to examine the efficacy of early initiation of breastfeeding, early skin-to-skin contact, and rooming-in for the continuation of EBF through to 6 months postpartum, and moreover this study aims to contribute to increasing the rate of EBF up to 6 months postpartum by identifying factors that sustain and inhibit EBF.

## Materials and methods

### Study population

**Figure 1:**
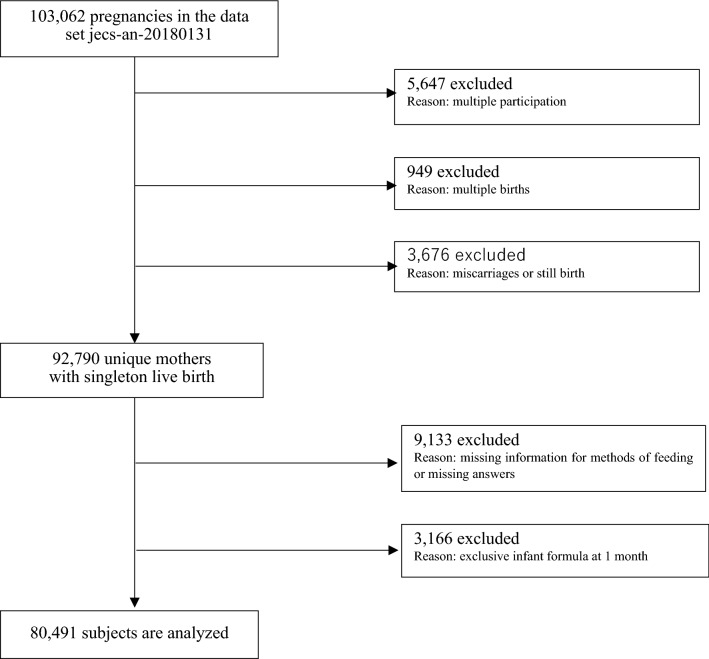
Participants flow diagram. See text for details.

JECS is a nationwide government-funded birth cohort study that evaluates the impact of various environmental factors on children’s health and development. The pregnant women participating in JECS were recruited from 15 areas in Japan between January 2011 and March 2014^10,11^. Further information on the methods utilised within JECS have been previously published^10,11^. The present study is based on a dataset (jecs-an-20180131) that was released in March 2018. The full dataset contained 103,062 pregnancies. Of these, 5,647 data points were excluded because of multiple registrations, 949 were excluded because of multiple births, and 3676 were excluded because of miscarriages or stillbirths. Among the remaining 92,790 unique mothers with singleton live births, 9133 were excluded because of missing information for methods of feeding or missing answers and 3166 were excluded because of the exclusive use of infant formula at one month. As a result, data from 80,491 mothers were analysed in this study (Fig. 1).

### Materials

The JECS self-administered questionnaire is an instrument provided to enrolled mothers and their partners during the first trimester and second/third trimester of pregnancy. One month after birth, a questionnaire is answered by the mothers and/or partners. Thereafter, questionnaires are administered every six months. Each questionnaire contains a section designated to collect information about chemical exposure, socioeconomic status, lifestyle factors, and physical environment^10^.

### JECS data items used

Data from medical record transcription forms were acquired for the following variables: maternal age, parity, fertilisation method, delivery method, presence or absence of obstetric complications, BMI before pregnancy (less than 18.5 kg/m^2^, 18.5 to 25 kg/m^2^, greater than 25 kg/m^2^), gestational period of the child, gender of the child, presence or absence of birth defects, and presence or absence of analgesia use during delivery. In addition, the cut-off point for obesity in this study, that is, BMI > 25 kg/m^2^ was the same as that in the profile paper^11^.

Daily caloric intake in the second/third trimester of pregnancy was determined by the Food Frequency Questionnaire, which is semi-quantitative and has been validated for use in large-scale Japanese epidemiologic studies^12^. Data were taken from maternal self-administrated questionnaires for the following variables: child nutrition method (e.g., breast milk, mixed, or formula), marital status, drinking habits, smoking habits, exercise habits, presence or absence of second-hand smoke exposure, use of cellular phone or SMS messaging while nursing, presence or absence of early initiation of breastfeeding within one hour of birth, presence or absence of early skin-to-skin contact, presence or absence of rooming-in, depression as assessed by the Edinburgh Postnatal Depression Scale (EPDS) at 1 month postpartum^13^, presence or absence of negative emotions related to pregnancy, social connections, presence or absence of an opportunity to return to one’s parents, person responsible for household work, presence or absence of starting childcare by the age of 6 months, father’s participation in parenting, presence or absence of confidence in the surroundings, regional social capital, mother’s employment status, household income and education levels of mother and father.

Data were also taken from maternal self-administrated questionnaires at 1 month postpartum for the presence or absence of experiences where the child’s condition worsened while breastfeeding. Worsening of the child’s condition was defined as urticaria, skin changes, redness in and around the mouth, and pallor within an hour of breastfeeding.

Data were also taken from maternal self-administrated questionnaires at 6 months postpartum for the presence or absence of a pet in the household. Presence of a pet was considered to be a factor that would increase the burden on mothers and also affect breastfeeding.

### Measurements

#### Exclusive breastfeeding (EBF)

Mothers who indicated on the JECS self-administered questionnaire form at 6 months postpartum that they practiced continued, uninterrupted exclusive breastfeeding from the first month postpartum to the sixth were placed in the EBF group.

#### Early initiation of breastfeeding, early skin-to-skin contact, rooming-in

Mothers who responded with answer (1) to the following question on the JECS self-administered questionnaire at 1 month postpartum were regarded as having performed early initiation of breastfeeding: ‘When did you first hold your nipple in your child’s mouth? (1) Within 1 h of birth (2) More than one hour after birth (3) I have not yet had the chance to hold my nipple in my child’s mouth’.

Similarly, individuals who responded with answer (1) to the following question were regarded as having performed early skin-to-skin contact: ‘Did you hold your new-born baby in your arms soon after giving birth? (1) I held my baby so that our skin would touch. (2) I held my baby, but our skin did not touch. (3) I did not hold my baby soon after birth’.

Finally, we used the following question as a scale assessment of rooming-in: ‘In the facility in which you gave birth, how long were you able to stay in the same room as your new-born? (1) Almost no time (2) About a quarter of a day (3) About half of a day (4) About three-quarters of a day (5) We were nearly always together’.

### Regional social capital

Social capital is a characteristic—similar to trust, norms, and network—of social organisations that refers to their ability to induce cooperative action in pursuit of a common goal.

The total point value of the responses of pregnant women to the following two questions on the JECS self-administered questionnaire was used to measure social capital: ‘1. The people of my neighbourhood trust each other’ and ‘2. The people of my neighbourhood help each other’. Both questions were asked during the second/third trimester and had the following four potential responses: ‘(1) I believe so (1 point); (2) I would assume so (2 points); (3) I would not assume so (3 points) (4) I do not believe so (4 points)’. Social capital was divided into the following four categories according to the total point values measured: high social capital—2–3 points; medium social capital—4 points; low social capital—5–6 points; very low social capital—7–8 points^14^.

### Analysis

Multiple logistic regression model was used to analyse the data. SAS version 9.4 (SAS institute Inc., Cary, NC) was used for all analyses. Adjusted odds ratios were analysed for all 35 variables. The threshold for statistical significance was 5%, and we performed two-tailed tests. Risk indices have been listed with odds ratios and 95% confidence intervals in the results tables.

## Results

### Subject characteristics

The 80,491 individuals included in the study had an average age of 31.1 years (± 4.97 years). Of these, 43% were first-time mothers, 81.7% had a vaginal delivery, and 10.0% were obese. Of the total sample, 30,070 individuals (37.4%) continued EBF through to 6 months postpartum (EBF group). The average age of the EBF group was 30.9 years (± 4.74 years). In the EBF group, 36.3% were first-time mothers, 84.6% had a vaginal delivery, and 7.2% were obese.

Of the 35 items postulated to be factors affecting continuing EBF, statistically significant differences were found for 31 items.

### Early postpartum nursing prolonged the period of EBF

According to Table 1, the factors related to early postpartum mother–child care were early skin-to-skin contact, early initiation of breastfeeding, and rooming-in; these were extracted as continuation factors for EBF. Initiation of breastfeeding within one hour of birth contributed more strongly to the continuation of EBF than did initiation of breastfeeding more than one hour after birth. Rooming-in for 18–24 h also contributed to the continuation of EBF.

**Table 1:** Factors related to early postpartum mother–child care influencing EBF rates until 6 months postpartum in Japan.

	Total number (n)	Percent EBF to 6 months	Odds ratio	95% confidence interval	Adjusted odds ratio	95% confidence interval
**Factors related to early postpartum mother–child care**						
**Early skin-to-skin contact**^**a**^** (n = 79,822)**						
No early skin-to-skin contact	18,315	30.1	Reference		Reference	
Early skin-to-skin contact	46,349	41.5	1.647	(1.588–1.709)	1.233	(1.165–1.304)
Carried baby against clothes	15,158	33.7	1.181	(1.127–1.236)	0.994	(0.932–1.059)
**Rooming-in**^**a**^** (n = 79,693)**						
Mother and child separated	5919	26.3	Reference		Reference	
Roomed-in for one-quarter of a day	4863	29.0	1.145	(1.052–1.246)	0.923	(0.834–1.022)
Roomed-in for half a day	7853	29.8	1.188	(1.102–1.281)	1.002	(0.915–1.098)
Roomed-in for three-quarters of a day	10,781	32.7	1.356	(1.264–1.455)	1.099	(1.009–1.198)
Roomed-in all day	50,277	41.8	2.008	(1.890–2.133)	1.567	(1.454–1.690)
**Early nursing**^**a**^** (n = 79,729)**						
Not nursed until 1 mo. postpartum	380	16.1	0.409	(0.311–0.538)	0.637	(0.410–0.990)
Within 1 h of birth	35,050	44.7	1.732	(1.682–1.783)	1.455	(1.401–1.512)
After 1 h of birth	44,299	31.9	Reference		Reference	

Data acquisition.

^a^1 month postpartum.

### The child started childcare by the age of 6 months shortened the period of EBF

Table 2 shows that the following social factors possibly affected continuation of EBF: presence or absence of confidence in the surroundings, regional social capital, degree of second-hand smoke exposure, whether one’s child had started childcare by 6 months of age, whether there was a return to one’s parents’ house, degree of paternal participation in parenting and who in the household did most of the housework.

**Table 2:** Environmental factors influencing EBF rates until 6 months postpartum in Japan.

	Total number (n)	Percent EBF to 6 months	Odds ratio	95% confidence interval	Adjusted odds ratio	95% confidence interval
**Child-related factors**						
**Gender of the child**^**d**^** (n = 80,491)**						
Female	39,254	38.2	Reference		Reference	
Male	41,237	36.6	0.935	(0.909–0.962)	0.964	(0.932–0.997)
Gestational period^d^ (in weekly intervals)	80,491		1.067	(1.056–1.077)	1.044	(1.029–1.059)
**Birth defects in the child**^**d**^** (n = 80,491)**						
Absent	78,850	37.6	Reference		Reference	
Present	1641	27.7	0.638	(0.572–0.711)	0.764	(0.669–0.873)
**Social factors**						
**Confidence**^**a**^** (n = 79,490)**						
Never available	1692	37.1	Reference		Reference	
Rarely available	5902	32.0	0.797	(0.712–0.892)	0.864	(0.755–0.990)
Somewhat available	15,960	34.3	0.884	(0.797–0.981)	0.874	(0.771–0.989)
Nearly always available	8202	37.0	0.996	(0.894–1.110)	0.912	(0.802–1.039)
Always available	47,734	39.2	1.094	(0.990–1.210)	0.983	(0.872–1.109)
**Regional social capital**^**a**^** (n = 78,820)**						
Extremely low	16,462	34.5	Reference		Reference	
Low	22,392	35.5	1.043	(1.000–1.088)	0.992	(0.943–1.043)
Medium	31,489	39.5	1.235	(1.187–1.284)	1.084	(1.033–1.138)
High	8477	41.2	1.328	(1.259–1.402)	1.133	(1.061–1.210)
**Second-hand smoke**^**b**^** (n = 79,921)**						
Absent	38,928	39.5	Reference		Reference	
Smoked outside of the presence of the baby	39,192	35.6	0.845	(0.821–0.870)	0.952	(0.917–0.988)
Smoked in the presence of the baby	1801	31.4	0.699	(0.632–0.774)	0.906	(0.797–1.029)
**Child attends childcare at 6 months**^**c**^** (n = 80,345)**						
Absent	74,919	39.5	Reference		Reference	
Present	5426	8.4	0.14	(0.127–0.155)	0.126	(0.113–0.141)
**Returning to one’s parents’ house**^**b**^** (n = 79,614)**						
Did not return to parents’ house	30,608	38.2	Reference		Reference	
Returned home for no more than 1 month	18,457	39.2	1.039	(1.001–1.079)	1.032	(0.985–1.081)
Returned home for longer than 1 month	20,871	36.6	0.930	(0.897–0.965)	0.930	(0.881–0.981)
Live together	9678	33.5	0.815	(0.776–0.855)	0.795	(0.746–0.848)
**Did you send your child to a nursery?**^**b**^** (n = 79,193)**						
No	61,110	39.0	Reference		Reference	
Yes	18,083	32.3	0.748	(0.722–0.774)	0.712	(0.683–0.743)
**Paternal participation in parenting**^**b**^** (n = 79,009)**						
No participation	2490	37.8	Reference		Reference	
Participates often	30,455	35.8	0.914	(0.840–0.995)	0.852	(0.769–0.945)
Participates occasionally	36,983	38.1	1.012	(0.931–1.100)	0.921	(0.832–1.018)
Almost never participates	9081	41.1	1.147	(1.047–1.257)	1.043	(0.935–1.163)
**Person doing most of the housework**^**b**^** (n = 79,919)**						
Mother herself	35,168	38.0	Reference		Reference	
Father	4023	35.1	0.885	(0.827–0.948)	0.961	(0.886–1.043)
Siblings	228	27.6	0.625	(0.467–0.835)	0.794	(0.547–1.153)
Maternal grandmother	33,749	37.5	0.983	(0.953–1.014)	0.971	(0.928–1.015)
Maternal grandfather	364	31.3	0.746	(0.597–0.932)	0.739	(0.565–0.967)
Paternal grandmother	5219	36.8	0.953	(0.897–1.012)	0.928	(0.859–1.001)
Paternal grandfather	55	36.4	0.934	(0.539–1.619)	0.799	(0.415–1.536)
Other, not listed above	1113	31.6	0.756	(0.665–0.860)	0.854	(0.731–0.999)

Data acquisition.

^a^During mid-pregnancy/ ^b^1 month postpartum/ ^c^6 months postpartum/ ^d^attending physician diagnosed just after delivery.

Among the 35 factors examined, the child starting childcare by the age of 6 months was found to be the most inhibiting. ‘Rarely available’ and ‘somewhat available’ confidences as compared to the absence of a confidence, were also inhibitory factors for EBF continuation. Further, close relatives’ smoking outside of the baby’s presence was an inhibiting factor. With regard to return to one’s parents’ house, a return for longer than 1 month or situations in which the mother lived with her parents were found to inhibit continuation of EBF. The greater the father’s participation in parenting, the more inhibitory it was for EBF continuation. Finally, situations in which the maternal grandfather was primarily responsible for housework were found to be inhibitory for EBF. The only social factor found to contribute to EBF continuation was medium to high regional social capital.

### Full term and female babies were more likely to be breastfed

According to Table 2, the following child related variables were found to support EBF continuation: gender, gestational period, and presence or absence of birth defects. Males were less likely than females to be exclusively breastfed to 6 months. The presence of birth defects was also found to be an inhibitory factor of EBF continuation. Additionally, the longer a child’s gestational period (measured in weeks), the more it contributed to the continuation of EBF.

### Negative social demographic factors obstructed EBF

Table 3 shows that the following social demographic factors contributed to the continuation of EBF: maternal age, maternal education level, paternal education level, maternal employment status, and parental marital status. The higher a mother’s age was (to the nearest year), the more it contributed to the inhibition of EBF continuation. With regard to maternal education level, mothers that graduated from vocational school, community college or a university were more likely to continue EBF. Paternal education level was associated in the same way with EBF. Mothers who were housewives and on leave from work were more likely to continue EBF than full-time employed mothers. Finally, divorce from or death of the husband was an inhibitory factor of EBF continuation compared to being married.

**Table 3:** Personal factors influencing EBF rates until 6 months postpartum in Japan.

	Total number (n)	Percent EBF to 6 months	Odds ratio	95% confidence interval	Adjusted odds ratio	95% confidence interval
**Social demographic factors**						
Age of the mother^e^ (by year)	79,950		0.984	(0.981–0.987)	0.957	(0.953–0.961)
**Household income**^**b**^** (n = 74,424)**						
Less than 4 million yen	29,078	35.9	Reference		Reference	
4–6 million yen	24,858	38.5	1.117	(1.079–1.157)	1.022	(0.980–1.066)
Greater than 6 million yen	20,488	39.3	1.156	(1.114–1.199)	1.049	(0.998–1.102)
**Mother’s level of education**^**b**^** (n = 79,533)**						
Middle/High school graduate	27,207	32.7	Reference		Reference	
Vocational school/ Community college graduate	34,396	38.4	1.285	(1.243–1.328)	1.188	(1.138–1.240)
College/University graduate or higher	17,930	42.7	1.535	(1.476–1.596)	1.322	(1.253–1.395)
**Father’s level of education**^**b**^** (n = 79,066)**						
Middle/High school graduate	33,685	34.1	Reference		Reference	
Vocational school/Community college graduate	18,153	38.6	1.213	(1.169–1.259)	1.079	(1.031–1.129)
College/University graduate or higher	27,228	41.0	1.342	(1.298–1.387)	1.104	(1.057–1.154)
**Mother’s employment status**^**b**^** (n = 79,277)**						
Full‐time	25,313	36.8	Reference		Reference	
Self‐employed or its assistant	2765	35.8	0.956	(0.881–1.038)	1.074	(0.972–1.186)
Temporary staff	1038	30.2	0.741	(0.648–0.849)	0.863	(0.733–1.015)
Housewife or on leave	34,696	40.4	1.163	(1.125–1.202)	1.045	(1.000–1.092)
Part‐time/Side job/Commission	12,890	33.1	0.848	(0.811–0.887)	0.954	(0.902–1.009)
Unemployed	1035	29.7	0.724	(0.632–0.830)	0.927	(0.777–1.107)
Other	1540	31.5	0.790	(0.707–0.882)	0.852	(0.746–0.972)
**Marital status**^**a**^** (n = 79,685)**						
Married	76,349	37.8	Reference		Reference	
Unmarried	2751	30.4	0.718	(0.661–0.780)	0.997	(0.889–1.119)
Divorced/widowed	585	23.3	0.500	(0.412–0.606)	0.683	(0.523–0.892)
**Maternal lifestyle factors**						
Daily calorie intake^b^ (per 1000 kcal)	79,951		1.020	(1.001–1.039)	0.995	(0.971–1.019)
**Drinking habits**^**b**^** (n = 79,355)**						
None	26,513	38.3	Reference		Reference	
Stopped before pregnancy	13,069	39.8	1.068	(1.023–1.115)	1.039	(0.988–1.094)
Stopped after discovering pregnancy	37,621	36.1	0.910	(0.881–0.940)	0.967	(0.930–1.006)
Continued during pregnancy	2152	36.3	0.918	(0.838–1.006)	0.995	(0.892–1.110)
**Smoking habits**^**c**^** (n = 79,851)**						
None	47,750	39.5	Reference		Reference	
Stopped before pregnancy	18,092	38.5	0.960	(0.927–0.995)	1.016	(0.973–1.061)
Stopped after discovering pregnancy	11,361	30.3	0.666	(0.637–0.696)	0.796	(0.751–0.842)
Continued during pregnancy	2648	22.1	0.434	(0.395–0.477)	0.557	(0.496–0.627)
**Exercise habits**^**b**^** (n = 79,704)**						
No exercise habits	19,189	35.4	Reference		Reference	
At least 10 min of exercise (walking) weekly	60,515	38.1	1.12	(1.082–1.158)	1.074	(1.031–1.118)
**Household pet**^**d**^** (n = 80,194)**						
Absent	60,435	38.6	Reference		Reference	
Present	19,759	33.9	0.817	(0.79–0.845)	0.907	(0.870–0.946)
**Factors related to maternal behavior during nursing**						
**Experience of child’s condition worsening during breastfeeding**^**c**^** (n = 79,200)**						
Absent	77,568	37.5	Reference		Reference	
Present	1632	39.9	1.107	(1.002–1.224)	1.144	(1.014–1.290)
**Activities of the mother during nursing**^**c**^** (n = 79,143)**						
Looked into the eyes of and talked with child	57,340	36.7	Reference		Reference	
Often watch TV or a DVD	12,299	37.0	1.014	(0.974–1.056)	0.954	(0.910–1.001)
Reading newspapers or magazines	510	44.7	1.394	(1.170–1.662)	1.130	(0.924–1.381)
Used cellphones or a PC	6037	40.0	1.148	(1.087–1.212)	1.101	(1.031–1.175)
Did housework	74	18.9	0.405	(0.227–0.724)	0.420	(0.203–0.868)
Something other than the above	2883	46.5	1.500	(1.391–1.617)	1.136	(1.039–1.242)

Data acquisition.

^a^During early pregnancy/ ^b^during mid-pregnancy/ ^c^1 month postpartum/ ^d^6 months postpartum/ ^e^attending physician diagnosed just after delivery.

### Having a household pet reduced the period of EBF

Based on Table 3, the following maternal lifestyle factors were found to be involved in the continuation of EBF: smoking habits, exercise habits, and having a household pet. Smoking was found to be an inhibitory factor of EBF continuation, even if the mother stopped smoking after discovering pregnancy. Engaging in a minimum of 10 min per week of light exercise (e.g., walking) was found to be a supporting factor of EBF continuation. Finally, the presence of a household pet was associated with a lower prevalence of EBF continuation. Each factor was tested for multicollinearity, and domestic violence was removed from the results because of the multicollinear relationship between smoking and domestic violence.

### EBF mothers were more likely to have looked at a computer or cell phone while breastfeeding

Table 3 shows experience of the child’s condition worsening during breastfeeding, and the use of one’s mobile phone or PC during nursing were found to support the continuation of EBF. On the other hand, housework during nursing was found to be an inhibitory factor of EBF continuation.

### Obesity in pregnancy reduced the period of EBF

Table 4 shows that the following maternal physical factors were found to influence EBF continuation: BMI and Parity. A BMI of 25 or greater indicating obesity was an inhibitory factor for EBF continuation. Multiparity was found to support EBF continuation.

**Table 4:** Maternal physical, psychological and medical factors influencing EBF rates until 6 months postpartum in Japan.

	Total number (n)	Percent EBF to 6 months	Odds ratio	95% confidence interval	Adjusted odds ratio	95% confidence interval
**Maternal physical factors**						
**BMI**^**c**^** (n = 80,435)**						
18.5–25 kg/m^2^	59,392	38.5	Reference		Reference	
Less than 18.5 kg/m^2^	13,033	38.6	1.005	(0.966–1.045)	0.983	(0.938–1.030)
Greater than 25 kg/m^2^	8010	27.0	0.592	(0.562–0.623)	0.667	(0.627–0.710)
**Parity**^**c**^** (n = 78,507)**						
Primipara	33,725	31.7	Reference		Reference	
Multipara	44,782	41.8	1.550	(1.505–1.597)	1.812	(1.737–1.891)
**Obstetrical complications**^**c**^** (n = 79,874)**						
Absent	68,071	37.7	Reference		Reference	
Present	11,803	35.4	0.905	(0.869–0.943)	1.028	(0.979–1.079)
**Maternal psychological factors**						
**Edinburgh scale score**^**b**^** (n = 79,000)**						
Less than 9	68,200	38.9	Reference		Reference	
Greater than or equal to 9	10,800	28.3	0.622	(0.595–0.651)	0.716	(0.678–0.756)
**Negative emotions towards pregnancy**^**a**^** (n = 79,655)**						
Absent	74,029	37.8	Reference		Reference	
Present	5626	32.1	0.778	(0.734–0.824)	0.906	(0.844–0.972)
**Medical factors**						
**Delivery method**^**c**^** (n = 80,352)**						
Vaginal delivery	65,672	38.7	Reference		Reference	
Caesarean section	14,680	31.5	0.730	(0.702–0.758)	1.161	(1.092–1.233)
**Analgesics during delivery**^**c**^** (n = 79,069)**						
Absent	77,177	37.6	Reference		Reference	
Present	1892	29.0	0.678	(0.613–0.749)	0.814	(0.723–0.916)
**Infertility treatments**^**a**^** (n = 79,649)**						
Absent	72,276	38.0	Reference		Reference	
Present	7373	31.3	0.744	(0.706–0.783)	0.853	(0.802–0.908)

Data acquisition.

^a^During early pregnancy/^  b^1 month postpartum/^   c^attending physician diagnosed just after delivery.

### An unsettled state of mind in pregnancy reduced the period of EBF

According to Table 4, the following maternal psychological factors were found to be inhibitory toward EBF continuation: negative emotions toward pregnancy and a score of 9 or greater on the EPDS.

### Medical intervention during the perinatal period tended to restrict EBF

Based on Table 4, the following medical factors were found to be involved in EBF continuation: delivery method, use of analgesics during delivery, and engagement in infertility treatments. Caesarean section delivery (more so than vaginal delivery) tended to be positively associated with EBF at 6 months. Analgesic use during delivery and infertility medications were found to be inhibitory factors of EBF continuation.

## Discussion

This large-scale cohort study clarified the factors involved in EBF continuation through to 6 months postpartum. In particular, the early initiation of breastfeeding—within one hour of birth—was shown to contribute greatly. Many previous studies^15^ have shown that early initiation of breastfeeding and early skin-to-skin contact are the keys to successful breastfeeding, and this study showed similar results. In addition, previously published research has indicated various factors as being effective in promoting the continuation of EBF including rooming-in^16,17^, education level^18^, presence of a child supporter^19^ and Parity^20^ and so on. In this study, as in the previous studies, the above factors were found to contribute to breastfeeding.

The higher the rate of EBF at 6 months, the greater the benefit to maternal and infant health and the greater the significance from a public health perspective. Therefore, the Global Breastfeeding Collective of UNICEF/WHO aims to increase the percentage of babies under 6 months old exclusively breastfed from 44^21^ to 70% by 2030^22^. The prevalence of exclusive breastfeeding in Japan at 3 months is 54.7%^4^ and at 6 months is 37.4%. These percentages are not high. However, the results of this study have provided several hints to increase the percentage of EBF.

The results of the present study revealed newly discovered factors influencing the continuation of EBF, such as regional social capital during pregnancy and ownership of a household pet. High regional social capital tends to associate with high levels of health among the residents of the region^23^. This study has revealed that high regional social capital was associated with a high breastfeeding rate, meaning that high regional social capital influences mother–child health. This discovery indicates that activities that seek to promote regional social capital may also have beneficial effects for maternal and child health.

Besides, previous research has shown that dog ownership has a positive effect on child development, so we considered it as a variable^24^. However, we found that pet ownership interfered with breastfeeding. Raising pets increases the burden on mothers, which results in mothers spending less time with their children. This also reduces the amount of time spent slowly feeding breast milk, which may have been extracted as a factor in reducing breastfeeding rates.

Regarding other factors, as reported in previously published research, our study found that the mother’s education level is positively associated with the continuation of EBF. According to literature, mothers who decide to engage in EBF inform themselves to a greater degree about nutrition and diet, consult more frequently with their doctors, and have better access to information on the health of nursing children than mothers who do not engage in EBF^25^. It may be the case that the higher a mother’s education level, the more aware she is of the merits of EBF and, therefore, her motivation to implement it is higher.

Regarding support in the parenting process, our research indicated that the father’s participation in parenting is negatively associated with continued EBF. This runs contrary to associations reported by other researchers^26^. It is very common for parents to supplement milk with a baby bottle, and this situation can make it easy for fathers to help with feeding. Therefore, it is thought that bottle feeding resulted in a higher rate of father participation in childcare, including with feeding. Nevertheless, in the context of the continuation of EBF, the importance of a childcare supporter has been made clear by previously published work^27^.

Regarding activities while nursing, it is generally believed that looking into the eyes of the child is beneficial from the perspective of forming an attachment with the child^28^, but the results of this study, there was no association between looking into the eyes of the child and the duration of EBF. Instead, we saw a trend that mothers who breastfed while looking at the newspaper or a mobile phone continued to EBF for longer. This suggests that for mothers who continue to EBF, breastfeeding itself is as integrated into their lives as looking at the newspaper or mobile phone. Since one hand is free while breastfeeding, simple actions that can be done with one hand can be done at the same time. However, for parents who are mixed feeding, it becomes difficult to look at the newspaper or a phone while feeding because both hands are required (to hold the baby and to hold the bottle). This may explain why the percentage of the EBF group who looked at a newspaper or mobile phone with one free hand while breastfeeding was higher than that for the mixed feeding group.

In this study, Caesarean sections were found to be positively associated with EBF at 6 months, which is inconsistent with the direction of association reported in most other studies. Many studies examining Caesarean sections and breastfeeding rates have examined breastfeeding rates less than 3 months postpartum as an endpoint^29^, but our study examined a relatively long breastfeeding period, with 6 months as an endpoint. The results of this study suggest that the period during which Caesarean section interferes with breastfeeding may be a relatively short period after delivery. In other words, mothers who gave birth by Caesarean section are likely to have difficulties in breastfeeding during the first 3 months after delivery, but after 3 months, they may be able to continue breastfeeding and the fact that they have undergone a Caesarean section does not interfere with EBF.

Reports on factors involved in the inhibition of EBF continuation include the following: low household income^30^, obesity^31,32^, engagement in smoking^33^, presence of domestic violence^34^, low birth weight^35^, use of epidural anaesthetics^36^ and a high EPDS score^37^. These factors were found to inhibit EBF in our research as well.

Other factors found to inhibit EBF continuation in this study were that the child started childcare, mother smoking, and obesity in early pregnancy. Among these, the child starting childcare by the age of 6 months or younger was found to inhibit EBF continuation most profoundly. Previously published research has indicated that even if a mother is employed full-time—a factor known to affect breastfeeding—giving one’s child over to another’s care before 6 months of age is associated with the cessation of breastfeeding^37,38^. The primary reason that necessitates the handing over of one’s child to another’s care is a mother’s need to return to her workplace^39^. The Japanese maternity leave system offers 8 weeks after giving birth, with the possibility of focusing on childcare for up to 1 year with some degree of paid salary if the mother is able to obtain childcare leave. The International Labour Organization recommends providing mothers with at least 18 weeks of maternity leave and 100% payment of salary during that period^40^, but this practice is yet to be enforced within Japan^41^. The child starting childcare makes the continuation of EBF quite difficult; mothers should receive at least 6 months of maternal leave. Alternatively, arrangements ought to be made to allow mothers who return to work before 6 months postpartum to bring their children with them to the workplace. This is because children that are breastfed rely exclusively on breast milk for their nutrition and require frequent feeding. Besides, many nurseries do not accept breast milk for hygiene reasons in Japan^42^. This makes exclusive breastfeeding difficult. Furthermore, the main reason for starting childcare is the mother's return to work. While a mother's return to work is not in itself an impediment to breastfeeding, the fact that she will no longer be able to breastfeed as often as she has been able to in the workplace is a problem for her to continue breastfeeding.

The potential implications of providing new mothers with an environment in which they can respond to their infants’ needs quickly include the rise of birth rates. Society at large—not just mothers and children—will benefit greatly from the windfall of such a change. If Japan truly intends to bring its EBF rate in line with the global benchmark, it is necessary to improve early postpartum care and overhaul the current maternity leave system.

There were several limitations in our research. First, the questions polling about initiation of breastfeeding within one hour of delivery, early skin-to-skin contact, and mother–child rooming-in were asked one month after birth; the answers were therefore subject to errors in recall, and their accuracy cannot be guaranteed. Similarly, mothers were asked to declare whether they engaged in EBF 6 months postpartum—they needed to look back on 6 months of childcare and remember their habits. This, too, may have introduced some unreliability into our data. However, because the number of cases we examined is quite large, the unreliability of a few answers should not affect our results in any meaningful way. Second, our participants were all mothers living in Japan; thus, generalising our findings to the global situation is difficult. Third, because the answers to the factor items (the independent variables) and the answers reporting EBF (the dependent variable) were collected at different times, caution regarding their cause-and-effect relationship is essential to the proper interpretation of our results. Finally, because this was a large-scale survey, it is possible that some spurious findings were discovered.

In conclusion, early postpartum mother–child care—initiation of breastfeeding within one hour of birth, early skin-to-skin contact, and rooming-in—was found to contribute greatly to the continuation of EBF till 6 months postpartum. In addition, high social capital was found to effectively promote continuation of EBF. On the other hand, the child starting childcare was found to greatly inhibit continuation of EBF. Hence, these findings suggest a necessity to provide a minimum of 6 months maternity leave and to provide mother–child support in care protocols in order to raise the rate of Japanese EBF during 0–5 months of age up to 70%.

### Ethics approval

The study protocol was approved by the Ministry of the Environment’s Institutional Review Board on Epidemiological Studies and by the Ethics Committee of all participating institutions. Written informed consent was obtained from all participants.
